# Development and validation of a new risk scoring system for solid tumor patients with suspected infection

**DOI:** 10.1038/s41598-022-07477-w

**Published:** 2022-03-02

**Authors:** Bora Chae, Seonok Kim, Yoon-Seon Lee

**Affiliations:** 1 Department of Emergency Medicine, University of Ulsan College of Medicine, Asan Medical Center, 88, Olympic-ro 43-gil, Songpa-gu, Seoul, 05505 Korea; 2 Department of Clinical Epidemiology and Biostatistics, University of Ulsan College of Medicine, Asan Medical Center, Seoul, Korea

**Keywords:** Diseases, Cancer, Cancer models

## Abstract

This study aimed to develop a new prognostic model for predicting 30-day mortality in solid tumor patients with suspected infection. This study is a retrospective cohort study and was conducted from August 2019 to December 2019 at a single center. Adult active solid tumor patients with suspected infection were enrolled among visitors to the emergency room (ER). Logistic regression analysis was used to identify potential predictors for a new model. A total of 899 patients were included; 450 in the development cohort and 449 in the validation cohort. Six independent variables predicted 30-day mortality: Eastern Cooperative Oncology Group (ECOG) performance status (PS), peripheral oxygen saturation (SpO_2_), creatinine, bilirubin, C-reactive protein (CRP), and lactate. The C-statistic of the new scoring system was 0.799 in the development cohort and 0.793 in the validation cohort. The C-statistics in the development cohort was significantly higher than those of SOFA [0.723 (95% CI: 0.663–0.783)], qSOFA [0.596 (95% CI: 0.537–0.655)], and SIRS [0.547 (95% CI: 0.483–0.612)]. The discriminative capability of the new cancer-specific risk scoring system was good in solid tumor patients with suspected infection. The new scoring model was superior to SOFA, qSOFA, and SIRS in predicting mortality.

## Introduction

Patients with cancer are susceptible to infection, which can lead to poor outcomes. In a recent cohort study of 1 million sepsis hospitalizations in the United States, one in five cases was associated with malignancy, and in-hospital mortality was higher in cancer-related sepsis hospitalizations (27.9% vs. 19.5% in non-cancer-related sepsis)^1^. The vulnerability of these patients to infection is driven by many factors, including their immunocompromised state caused by anti-cancer treatments, frequent use of broad-spectrum antibiotics, and indwelling catheters^2,3^. Malnutrition caused by disruption of mucosal integrity and insufficient oral intake can also aggravate the immunosuppressive condition of these patients^4^. Therefore, it is critical to recognize the severity of their condition and promptly provide appropriate treatment.

The clinical presentation of cancer patients with infection can differ from the typical signs and symptoms of infection alone. Immunomodulation can often prevent the onset of fever, even in severe cases of infection. Additionally, inflammatory markers can be elevated in both infectious and non-infectious patients with cancer^5^. Organ dysfunction indicators such as elevated levels of creatinine or bilirubin, confused mentality, or respiratory distress are also often chronically held in patients with cancer regardless of infection. Such altered and inconstant clinical features can lead to an inaccurate understanding of the severity of the patient’s condition and poor outcomes.

Existing severity scoring systems such as Systemic Inflammatory Response Syndrome (SIRS), Sequential Organ Failure Assessment (SOFA), and quick SOFA (qSOFA) have been used to predict the outcomes of critically ill patients^6,7^. However, while studies have reported that SOFA is superior to qSOFA and SIRS, there have been few reports of the accuracy of these scoring systems to assess patients with cancer^8,9^. There are few cancer-specific prognostic models that consider the characteristics of patients with cancer; therefore, to accurately risk-stratify these patients with suspected infection, a specialized, optimal prognostic model is needed. This study aimed to develop a new scoring system for predicting mortality in cancer patients with suspected infection.

## Methods

### Study design and patients

This study was retrospectively conducted in the emergency room (ER) of a tertiary referral center in Seoul, South Korea. Patients who attended the ER between August 1, 2019 and December 31, 2019 and met the following criteria were included in the study: (1) aged ≥ 18 years, (2) had active solid cancer, and (3) were suspected of having an infection and needed intravenous (IV) antibiotic treatment by the physician^10–12^. Active cancer was defined as any of the following; 1) cancer that has been newly diagnosed within 6 months of study initiation; (2) receiving anti-cancer treatment; and (3) cancer that has progressed within the past 6 months^8^. All patients with suspected infection were performed with laboratory tests, blood and body fluid cultures, and imaging tests for detecting infection foci, and then administered IV antibiotics for therapeutic purposes.

Patients who were not suspected of infection or had low probability of infection were excluded. The ‘no suspected or low probability of infection’ refers to cases where blood cultures were not performed, antibiotics were not used, or antibiotics were used only for prophylactic purposes depending on the physician’s judgment. Patients who had hematologic malignancies, already used antibiotics before the ER arrival, lost at follow-up, did not have adequate workup at the ER, or refused even minimal life-sustaining treatment were excluded. For multiple visits during the study period, the information on the first visit was collected.

According to the time of ER visit, patients were divided into two groups: a development cohort from August 1, 2019 to September 30, 2019 and a validation cohort from October 1, 2019 to December 31, 2019. Any patient-identifying information was excluded from the study.

### Data collection and evaluation

Data were collected retrospectively from the hospital’s electronic medical records. Clinical variables included demographics, comorbidities, type of cancer, cancer stage, Eastern Cooperative Oncology Group (ECOG) performance status (PS)^13^, and initially measured vital signs, including mental status on arrival the ER. Comorbidities, including hypertension, diabetes mellitus, chronic renal disease, chronic liver disease, chronic lung disease, cardiovascular disease, and cerebrovascular disease, were analyzed based on the medical records at ER presentation. Chronic renal disease is defined as the glomerular filtration rate (GFR) has last less than 60 ml/min/1.73 m^2^ through 3 more months. Patients who could not check the previous GFR value were excluded. According to the Alert/responsive to Voice/responsive to Pain/Unresponsive (AVPU) scale, mental status was assessed. The AVPU values could be substituted to Glasgow Coma Scale (GCS) scores of 15, 13, 8, and 6, respectively^14^. A score of < 15 on the GCS was defined as an indication of altered mental status. Three sets of blood cultures were obtained before the administration of IV antibiotics during the ER stay; if patients had a catheter, one set of the three was drawn from the catheter. Laboratory data included complete blood count, chemistry, electrolytes, coagulation battery, inflammatory markers such as C-reactive protein (CRP), and serum lactate. The first obtained value was used in cases with multiple test results. For lactate, the values higher than 15.0 mmol/L were reported as ‘ > 15.0 mmol/L’. The SIRS, qSOFA, and SOFA scores were calculated based on the ER’s physiological and laboratory data.

### Statistical analysis

The primary outcome was 30-day mortality. Data were reported as the mean ± standard deviation (SD) or median and inter-quartile range for continuous variables. They were compared between groups using the Student’s *t*-test. Categorical variables were presented as the number and percentage and were compared using a chi-squared test.

To develop a new scoring system for 30-day mortality, univariate and multivariate logistic regression analyses were performed with an entering procedure in the development cohort. Results were summarized as odds ratios (OR) and respective 95% confidence intervals (CI). Variables with a *P*-value of < 0.1 in the univariate analysis and clinical relevance were considered for the multivariate analysis. A simple risk score was devised using the penalized maximum likelihood estimates of the predictors in the multivariable model. The constant of the scoring system was defined as one-third of the highest regression coefficient. The score was the weighted sum of those predictors. The weights were defined as the rounded integer value of the regression coefficients’ quotient value divided by the constant. The risk score’s discrimination capability was assessed using the C-statistic. Continuous variables were categorized in the new prediction model. The cutoff values of creatinine and total bilirubin were determined based on the normal values of our institution. The cutoff values of SpO_2_ (94%), lactate (2 mmol/L), and CRP (10 mg/dL) were set by referring to previous studies.

The calibration capability of the risk score was assessed by the Hosmer–Lemeshow test and calibration plot. Internal validation was performed by bootstrapping with 1000 iterations and calculated optimism-corrected C-statistic. The risk score was then categorized into low, intermediate, and high-risk groups based on the likelihood of 30-day mortality. A *P*-value of < 0.05 was considered statistically significant^15,16^.

The predictive performances of the SOFA, qSOFA, SIRS scores, and the new scoring system were analyzed by using the C-statistics values and compared with Delong’s test. A C-statistics of 1.0 denotes perfect, whereas a value close to 0.50 indicates no apparent accuracy. All statistical analyses were performed using SAS software version 9.4 (SAS Institute Inc., Cary, NC), R version 3.6.1 (R Foundation for Statistical Computing, Vienna, Austria, http://www.R-project.org), and IBM SPSS Statistics for Windows, version 21.0 (IBM Corp., Armonk, NY, USA).

### Ethics approval

This retrospective chart review study involving human participants was in accordance with the ethical standards of the institutional and national research committee and with the 1964 Helsinki Declaration and its later amendments or comparable ethical standards. The Institutional Review Board of Asan medical center approved this study.

### Consent to participate

Informed consent was waived by the Institutional Review Board of Asan medical center in view of the retrospective nature of the study and all the procedures being performed were part of the routine care.

## Results

### Baseline characteristics of the total population

**Figure 1:**
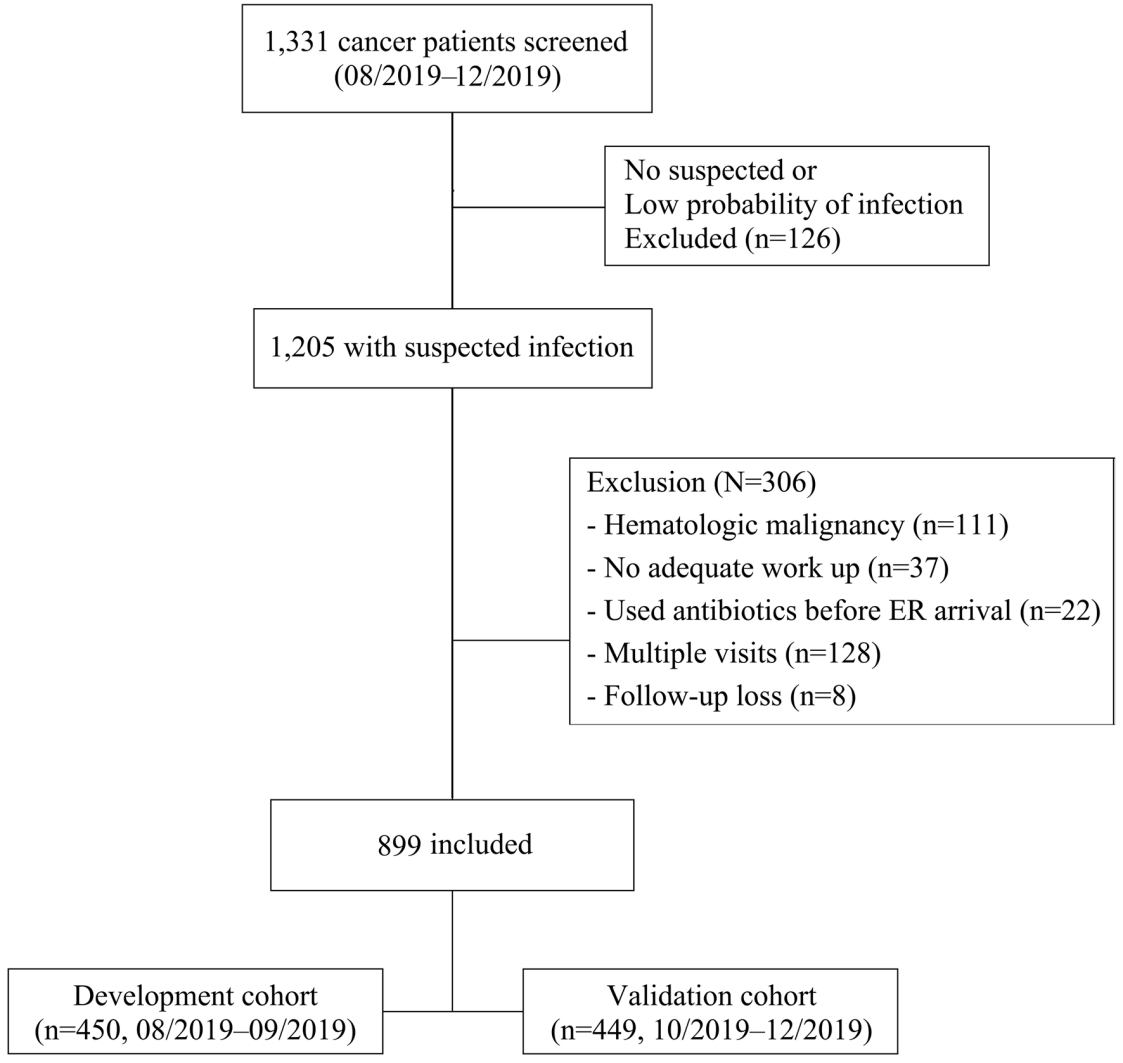
Flow chart. A total of 1331 patients with cancer were screened during the study period. First, patients with no suspected infection or low probability of infection (n = 126) were excluded. Among the remaining 1205 patients, 306 were excluded for the following reasons: 111 with hematologic malignancies, 37 with no adequate workup, 22 with used antibiotics before ER arrival, 128 with multiple visits, and 8 lost at follow-up. A total of 899 patients were included: 450 in the development cohort or 449 in the validation cohort. (Image created using Adobe Photoshop Version, 22.1.0 20201125.r.94 2020/11/25: 4b16c876033 × 64).

As shown in Fig. 1, 1331 cancer patients were initially screened at ER during the study period, and 126 with no suspected infection or low probability of infection were excluded. Among the remaining 1205 patients, 111 with hematologic malignancies, 37 with no adequate workup, 22 with used antibiotics before ER arrival, 128 with multiple visits, and 8 with follow-up loss were excluded additionally. A total of 899 patients were finally included in the study: 450 in the development cohort and 449 in the validation cohort.

Table 1 shows the baseline characteristics in total study subjects. The mean age was 63.0 (SD 11.8) years, and the male was 55.6%. The most common cancer was lung cancer (19.2%), followed by biliary cancer (16.1%) and pancreatic cancer (13.9%). Lung and hepatobiliary infections were the most common in 24.4% and 25.1%. The overall 30-day mortality was 22.5%. There was a significant difference in the 30-day mortality rate between development and validation cohorts (19.3% and 25.6%, P = 0.024).

**Table 1:** Baseline characteristics of total population.

	Total (*n* = 899)	Development *(n* = 450)	Validation (*n* = 449)	*P* value
Age (years)	63.0 ± 11.8	63. 7 ± 11.9	62.2 ± 11.7	0.073
Male, n (%)	500 (55.6)	262 (58.2)	238 (53.0)	0.116
**Cancer type, n (%)**				
Lung	173 (19.2)	99 (22.0)	74 (16.5)	0.276
Biliary	145 (16.1)	71 (15.8)	74 (16.5)	
Pancreas	125 (13.9)	66 (14.7)	59 (13.1)	
Breast	86 (9.6)	41 (9.1)	45 (10.0)	
Liver	73 (8.1)	40 (8.9)	33 (7.3)	
Gynecology	69 (7.7)	29 (6.4)	40 (8.9)	
Stomach	47 (5.2)	23 (5.1)	24 (5.3)	
Others^a^	181 (20.1)	81 (18.0)	100 (22.3)	
**Infection focus, n (%)**				
Lung	219 (24.4)	114 (25.3)	105 (23.4)	0.181
Hepatobiliary	226 (25.1)	122 (27.1)	104 (23.2)	
GI & intra-abdominal	89 (9.9)	43 (9.6)	46 (10.2)	
UTI	79 (8.8)	45 (10.0)	34 (7.6)	
Unknown	140 (15.6)	63 (14.0)	77 (17.1)	
Others^b^	146 (16.2)	63 (14.0)	83 (18.5)	
30-day mortality, n (%)	202 (22.5)	87 (19.3)	115 (25.6)	0.024

Values are expressed as the mean ± standard deviation and the number (%).

*GI* gastrointestinal, *UTI* urinary tract infection.

^a^Others in cancer type: esophagus, duodenum, small bowel, colon, rectum, head & neck, prostate, renal, bladder, thymoma.

^b^Others in infection focus: bone & soft tissue, bacteremia, febrile neutropenia.

### Comparison of characteristics between survivors and nonsurvivors

Table 2 shows the comparison of characteristics between survivors and nonsurvivors in 30 days in the development cohort. Nonsurvivors were older than survivors (mean age, 66.0 vs. 63.1 years, P = 0.040). Males were more frequent in non-survivors than in survivors (71.3% vs. 55.1%, P = 0.006). There was no significant difference in comorbidities, but the chronic renal disease was more common in nonsurvivors than survivors (8.05% vs. 0.8%, P < 0.001). For ECOG, nonsurvivors had poorer performance status than survivors (P < 0.001). Among vital signs, there was a significant difference in body temperature between nonsurvivors and survivors (37.1 °C vs. 37.6 °C, P < 0.001), and SpO_2_ tended to be lower in nonsurvivors (P = 0.05). Altered mental status was shown more frequently in nonsurvivors than in survivors (8.0% vs. 2.2%, P = 0.006). For laboratory data, there was a significant difference in WBC, Creatinine, CRP, and lactate levels between the two groups. Infection was documented through radiologic studies in 54.7% and body fluid cultures in 31.1%: blood in 16.6%, urine in 15.8%, and sputum in 13.3%. There was no significant difference in documented infection between survivors and nonsurvivors. Bacteremia was shown in 72 patients (16.6%) in the development cohort, and there was no difference between the two groups (15.4% vs. 18.4%, P = 0.498). Besides, multi drug resistant (MDR) bacteremia, respiratory virus, and fungal infection were identified in 32, 8, and 3 patients, respectively, showing no difference between survivors and nonsurvivors.

**Table 2:** Comparison of characteristics between survivors and non-survivors in 30 days in the development cohort.

	Total (*n* = 450)	Survivors *(n* = 363)	Non-survivors (*n* = 87)	*P* value
Age (years)*	63.7 ± 11.9	63.1 ± 12.0	66.0 ± 11.5	0.040
Male, n (%)	262 (58.2)	200 (55.1)	62 (71.3)	0.006
**Comorbidity, n (%)**				
Hypertension	148 (32.9)	116 (32.0)	32 (36.8)	0.390
Diabetes mellitus	276 (61.3)	220 (60.6)	56 (64.4)	0.518
Chronic renal disease	10 (2.2)	3 (0.8)	7 (8.0)	< 0.001
Chronic lung disease	33 (7.3)	23 (6.3)	10 (11.5)	0.097
Cardiovascular disease	257 (57.1)	207 (57.0)	50 (57.5)	0.940
Cerebrovascular disease	26 (11.9)	18 (10.3)	8 (18.6)	0.132
Chronic liver disease	262 (58.2)	212 (58.4)	50 (58.2)	0.874
Metastasis, n (%)	248 (55.1)	188 (51.8)	60 (69.0)	0.004
Anti-cancer treatment, n (%)	289 (64.2)	240 (66.1)	49 (56.3)	0.087
**ECOG PS, n (%)**				
0–1	136 (30.2)	129 (35.5)	7 (8.0)	< 0.001
2	220 (48.9)	174 (47.9)	46 (52.9)	
3–4	94 (20.9)	60 (16.5)	34 (39.1)	
Vital signs*				
SBP (mmHg)	117.5 ± 23.5	118.1 ± 22.7	114.6 ± 26.7	0.208
Heart rate (bpm)	103.8 ± 20.4	103.6 ± 20.3	105.0 ± 20.8	0.571
SpO_2_ (%)	96.1 ± 3.7	96.3 ± 3.4	95.3 ± 4.7	0.050
Body temperature (℃)	37.5 ± 1.1	37.6 ± 1.0	37.1 ± 0.9	< 0.001
Altered mental status, n (%)	15 (3.3)	8 (2.2)	7 (8.0)	0.006
Laboratory data*				
**WBC (× 10**^**3**^**/μL)**	9.9 ± 8.0	8.9 ± 6.4	14.2 ± 11.6	< 0.001
Hemoglobin (g/dL)	10.5 ± 2.0	10.5 ± 2.0	10.2 ± 2.1	0.166
Platelet (× 10^3^/μL)	209.7 ± 132.1	206.5 ± 122.2	223.0 ± 167.5	0.389
Creatinine (mg/dL)	1.08 ± 0.96	0.95 ± 0.71	1.63 ± 1.53	< 0.001
Total bilirubin (mg/dL)	2.0 ± 3.4	1. 8 ± 3.0	2.8 ± 4.7	0.054
CRP (mg/dL)	10.4 ± 10.9	9.0 ± 8.1	16.2 ± 17.6	< 0.01
Lactate (mmol/L)	2.0 ± 1.7	1.8 ± 1.4	2.7 ± 2.6	0.003
**Documented infection, n (%)**	394 (87.6)	325 (89.5)	69 (79.3)	0.009
Radiologic study^a^	246 (54.7)	206 (56.7)	40 (46.0)	0.070
Body fluid culture^b^	140 (31.1)	111 (30.6)	29 (33.3)	0.618
**Bacteremia**	72 (16.0)	56 (151.4)	16 (18.4)	0.498
Molecular assay^c^	13 (2.9)	12 (3.3)	1 (1.1)	0.281

Values are expressed as the mean ± standard deviation and the number (%).

*CRP* C-reactive protein, *ECOG* Eastern Cooperative Oncology Group, *PS* performance status, *SBP* systolic blood pressure, *SpO*_*2*_ peripheral oxygen saturation, *WBC* white blood cells.

^a^Infection was documented by radiologic studies, including chest x-ray, thorax or abdomen and pelvis computed tomography.

^b^Infection was documented by body fluid culture: blood in 16.0%, urine in 15.8%, and sputum in 13.3%.

^c^Infection was documented by molecular assay included polymerase chain reaction (PCR) for respiratory virus or antigen tests for Pneumococcus or Legionella, Aspergillus, etc.

### Logistic regression analysis for 30-day mortality in the development cohort

Univariate and multivariate logistic regression analyses of a 30-day mortality were performed as shown in Table 3. In the univariate regression, age (OR 1.02; 95% CI: 1.00–1.04), male sex (OR 2.02; 95% CI: 1.22–3.36), metastasis (OR 2.07; 95% CI: 1.26–3.41), chronic renal disease (OR 10.50; 95% CI: 2.66–41.49), ECOG PS 2 (OR 4.87; 95% CI: 2.13–11.14), ECOG PS 3–4 (OR 10.44; 95% CI: 4.38–24.91), SpO_2_ (OR 0.93; 95% CI: 0.87–0.99), altered mental status (OR 3.88; 95% CI: 1.37–11.02), creatinine (OR: 1.78; 95% CI: 1.41–2.24), total bilirubin (OR 1.07; 95% CI: 1.01–1.14), CRP (OR 1.07; 95% CI: 1.04–1.09), and lactate ≥ 2.0 mmol/L (OR 2.72; 95% CI: 1.69–4.39) were significantly associated with a 30-day mortality (P < 0.05 for all). In the multivariate regression analysis, ECOG PS 2 (OR 3.57; 95% CI: 1.60–7.96), ECOG PS 3–4 (OR 6.26; 95% CI: 2.67–14.71), SpO_2_ (OR 0.90; 95% CI: 0.84–0.97), creatinine (OR 1.57; 95% CI: 1.25–1.98), total bilirubin (OR 1.09; 95% CI: 1.02–1.16), CRP (OR 1.06; 95% CI: 1.03–1.09), and lactate ≥ 2.0 mmol/L (OR 2.58; 95% CI: 1.49–4.48) were independent predictors of 30-day mortality.

**Table 3:** Logistic regression analysis of the 30-day mortality in the development cohort.

	Univariate		Multivariate	
	OR (95% CI)	*P* value	OR (95% CI)	*P* value
Age	1.02 (1.00–1.04)	0.041	1.01 (0.98–1.03)	0.522
Male	2.02 (1.22–3.36)	0.007	1.46 (0.79–2.69)	0.232
Metastasis	2.07 (1.26–3.41)	0.004	1.39 (0.77–2.52)	0.274
Chronic renal disease	10.50 (2.66–41.49)	0.001	1.27 (0.21–7.69)	0.796
**ECOG PS**				
0–1	Reference		Reference	
2	4.87 (2.13–11.14)	< 0.001	3.57 (1.60–7.96)	0.002
3–4	10.44 (4.38–24.91)	< 0.001	6.26 (2.67–14.71)	< 0.001
SpO_2_	0.93 (0.87–0.99)	0.019	0.90 (0.84–0.97)	0.004
Altered mental status	3.88 (1.37–11.02)	0.011	1.45 (0.38–5.57)	0.589
Creatinine	1.78 (1.41–2.24)	< 0.001	1.57 (1.25–1.98)	< 0.001
Total bilirubin	1.07 (1.01–1.14)	0.019	1.09 (1.02–1.16)	0.017
CRP	1.07 (1.04–1.09)	< 0.001	1.06 (1.03–1.09)	< 0.001
Lactate ≥ 2 mmol/L	2.72 (1.69–4.39)	< 0.001	2.58 (1.49–4.48)	0.001

*CI* confidence interval, *CRP* C-reactive protein, *ECOG* Eastern Cooperative Oncology Group, *OR* odds ratio, *PS* performance status, *SpO*_*2*_ peripheral oxygen saturation.

### Development of a new risk scoring system

ECOG PS, SpO_2_, creatinine, total bilirubin, CRP, and lactate were selected for the new scoring system. Allocated points for each variable were as follows; 1 point for each SpO_2_ < 94%, creatinine ≥ 1.2 mg/dL, total bilirubin ≥ 1.2 mg/dL, CRP ≥ 10.0 mg/dL, 2 points for ECOG PS 2, lactate ≥ 2.0 mmol/L, and 3 points for ECOG PS 3–4. Finally, a 9-point risk scoring system with six variables was developed, as shown in Table 4. The calculation of the allocated point was described in Supplementary S.1.

**Table 4:** The new prognostic risk scoring system for cancer patients with suspected infection.

Variables	Regression coefficient	Score
ECOG PS 2	1.272	2
ECOG PS 3–4	1.835	3
SpO2 < 94%	−0.106	1
Creatinine ≥ 1.2 mg/dL	0.452	1
Total bilirubin ≥ 1.2 mg/dL	0.083	1
CRP ≥ 10.0 mg/dL	0.059	1
Lactate ≥ 2.0 mmol/L	0.949	2
Total score		9

*CRP* C-reactive protein, *ECOG* Eastern Cooperative Oncology Group, *PS* performance status, *SpO*_*2*_ peripheral oxygen saturation.

**Figure 2:**
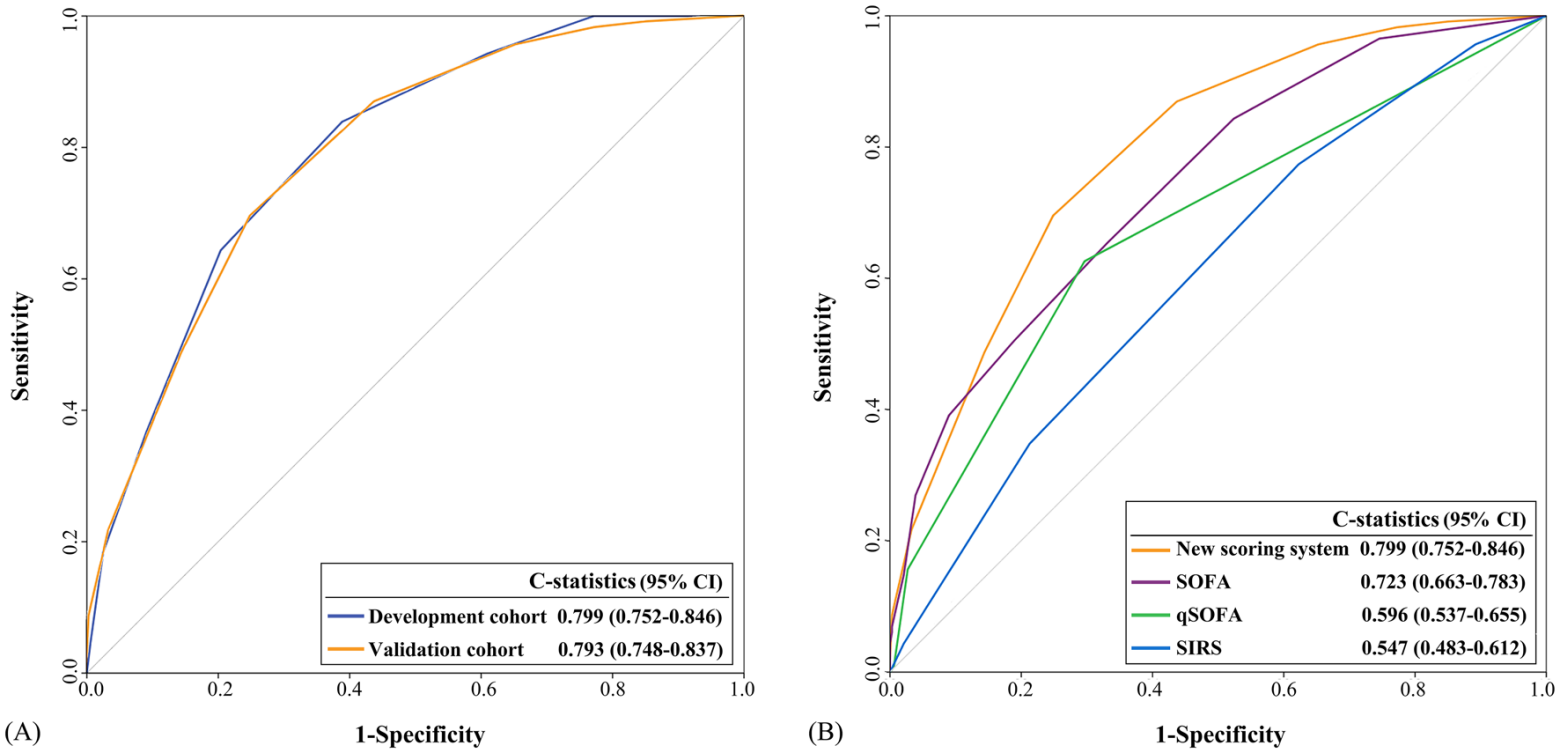
The C-statistics of the new scoring system in the development cohort and the validation cohort **(A)**, and comparison of the new scoring system with SOFA, qSOFA, and SIRS **(B)**. The C-statistics for the 30-day mortality of the new scoring system was 0.799 (95% confidence interval [CI]; 0.752–0.846) in the development cohort and 0.793 (95% CI; 0.747–0.838) in the validation cohort **(A)**. The C-statistics of the new scoring system was significantly higher than those for SOFA [0.723 (95% CI: 0.663–0.783, P = 0.018)], qSOFA [0.596 (95% CI: 0.537–0.655, P < 0.001)], and SIRS [0.547 (95% CI: 0.483–0.612, P < 0.001)]. *CI* confidence intervals, *qSOFA* quick sequential organ failure assessment, *SIRS* systemic inflammatory response syndrome, *SOFA* sequential organ failure assessment (Image created using Adobe Photoshop Version, 22.1.0 20201125.r.94 2020/11/25: 4b16c876033 × 64).

The C-statistic of the new scoring system was 0.799 (95% CI: 0.752–0.846) in the development cohort and 0.793 (95% CI: 0.748–0.837) in the validation cohort. For internal validation, the optimism-corrected C-statistic was 0.784 (95% CI: 0.737–0.831). The calibration graphs show that the new scoring system’s predicted and observed mortality risks in the development and validation cohorts were well-calibrated (Supplementary S.2). Additionally, the Hosmer–Lemeshow test did not indicate statistical significance for development (χ2 = 3.84, df = 6, P = 0.698) and validation cohorts (χ2 = 5.40, df = 6, P = 0.493). For predicting 30-day mortality in cancer patients with suspected infection, the new scoring system had a higher value of C-statistics than SOFA (0.723, 95% CI: 0.663–0.783, P = 0.018), qSOFA (0.596, 95% CI: 0.537–0.655, P < 0.001), and SIRS (0.547, 95% CI: 0.483–0.612, P < 0.001) (Fig. 2).

## Discussion

We have developed a new cancer-specific scoring system for solid cancer patients with suspected infection. This new system had a strong discriminative power to predict 30-day mortality, showing a C-statistic of 0.799 in the development cohort and 0.793 in the validation cohort. In comparison with existing scoring systems, the new scoring system was superior to SOFA (C-statistic, 0.723), qSOFA (C-statistic, 0.596), and SIRS (C-statistic, 0.547).

This new scoring system was developed and validated in two groups of similar size, recruited at different times in the same hospital. There was no significant difference in baseline characteristics between the two development and validation cohorts. In our study, the overall 30-day mortality rate was 22.5%. There was a difference in 30-day mortality between development and validation cohorts (19.3% vs. 25.6%, P = 0.024). However, the C-statistic (0.799 in the development cohort, 0.793 in the validation cohort) of our new scoring system showed a good discriminative capability to predict 30-day mortality in both groups. The Hosmer–Lemeshow goodness to fit test suggested that predicted mortality reflects true mortality, and thus our scoring system is well-calibrated. Several prognostic scoring systems have been used to predict prognosis in patients with infection^17–19^. SOFA is one of the most frequently validated systems and is an excellent predictor of mortality^20^. However, most of the SOFA studies were on non-cancer patients, and studies on those with cancer were limited with inconsistent results^8,9,21^. In a recent study, SOFA had good discriminative power in patients with cancer and was superior to qSOFA^8^. In two previous studies, both SOFA and qSOFA showed weak discriminative ability with a C-statistic of < 0.7 in predicting mortality for cancer patients with infection^9,21^. As shown in this study, our new scoring system can be a good alternative to predict mortality in cancer patients with suspected infection.

The new cancer-specific scoring system consisted of six components: ECOG PS, SpO_2_, creatinine, total bilirubin, CRP, and lactate. These six components reflect the underlying condition of patients with cancer and acute responses to infection. The ECOG PS of these patients has been considered as an essential prognostic factor^22,23^. ECOG PS had the highest score distribution among the new scoring system variables in our study. Performance status is affected by many factors such as the patients’ age, cancer stage, and side effects of anti-cancer treatment. Patients who have a poor PS and limited functional capacity tend to have more difficulty tolerating rigorous cancer treatments. These patients have less favorable outcomes than those with a better PS, regardless of distant metastasis or treatments given^24^. In this study, ECOG PS was a significant prognostic factor in cancer patients with suspected infection, whereas advanced stage or anti-cancer treatment were not. Lactate represents tissue hypoperfusion, and lactate > 2 mmol/L was introduced as diagnostic criteria of septic shock^25^. Furthermore, lactate has been shown to have prognostic power in cancer patients with sepsis^8^. Increased CRP is an indicator of inflammation in patients with sepsis. A recent study reported that CRP carries significant independent prognostic information^26^, which was consistent with the results of our study; elevated CRP was associated with increased 30-day mortality in cancer patients with suspected infection. Creatinine and total bilirubin are indicators of hepatic and renal function, which are also components of SOFA^27^. SOFA included PaO_2_/FiO_2_ as a respiratory indicator. We used SpO_2_ in place of PaO_2_/FiO_2_ given the ease to continuously and noninvasively obtain at the ER without drawing arterial blood. A previous study reported that SpO_2_ was consistently associated with mortality in patients with septic shock^28^.

This study has several limitations. First, it was conducted in a single hospital, which was a tertiary referral cancer center. There may have been a high proportion of severe disease. In our study, the 30-day mortality rate was 22.5%; however, in the US population report, cancer-related sepsis hospitalizations had high in-hospital mortality of 27.9%^1^. Second, as this study used a retrospective design, there were some limitations in data collection. However, we have a specialized area for cancer patients in ER, Cancer ER, ER for cancer patients^29^, and cancer patients are treated according to standardized protocols for their conditions and chief complaints. In this study, we tried to create a prognosis model with basic clinical variables included in the protocols for the treatment of patients with suspected infection. For this reason, we had very little missing data. Lastly, we included only solid tumors and excluded hematologic malignancies in this study. Therefore, it is difficult to apply the new scoring system to patients with hematologic malignancies. Many studies have shown that prognosis of patients with solid tumors and hematologic malignancies is quite different^30,31^. We consider that the prognostic model would have to be different to assess these two groups appropriately.

In conclusion, the new scoring system had a robust discriminative capability to predict prognosis in cancer patients with suspected infection. This new scoring system can be a good alternative for patients with solid tumor compared with existing scoring systems.

## Conclusion

A new risk scoring system in active cancer patients with suspected infection consisted of six components: ECOG PS, SpO_2_, creatinine, total bilirubin, CRP, and lactate. The new scoring system was superior to the existing scoring systems of SIRS, qSOFA, and SOFA in predicting 30-day mortality. We believe that our system can help physicians at the ER to predict prognosis for cancer patients more accurately and inform treatment decisions.

## Supplementary Information

Supplementary Information.

## Data Availability

The datasets generated during and/or analyzed during the current study are available from the corresponding author on reasonable request.

## References

[1] Hensley MK, Donnelly JP, Carlton EF, Prescott HC (2019). Epidemiology and outcomes of cancer-related versus non-cancer-related sepsis hospitalizations. Crit. Care Med..

[2] Jiang AM (2020). Nosocomial infections due to multidrug-resistant bacteria in cancer patients: A six-year retrospective study of an oncology Center in Western China. BMC Infect. Dis..

[3] Neuburger S, Maschmeyer G (2006). Update on management of infections in cancer and stem cell transplant patients. Ann. Hematol..

[4] Finn OJ (2012). Immuno-oncology: Understanding the function and dysfunction of the immune system in cancer. Ann. Oncol..

[5] Soares M, Feres GA, Salluh JI (2009). Systemic inflammatory response syndrome and multiple organ dysfunction in patients with acute tumor lysis syndrome. Clinics (Sao Paulo).

[6] Bone RC (1992). Definitions for sepsis and organ failure and guidelines for the use of innovative therapies in sepsis. The ACCP/SCCM Consensus Conference Committee. American College of Chest Physicians/Society of Critical Care Medicine. Chest.

[7] Angus DC (2016). A framework for the development and interpretation of different sepsis definitions and clinical criteria. Crit. Care Med..

[8] Chae BR, Kim YJ, Lee YS (2020). Prognostic accuracy of the sequential organ failure assessment (SOFA) and quick SOFA for mortality in cancer patients with sepsis defined by systemic inflammatory response syndrome (SIRS). Support Care Cancer.

[9] Costa RT, Nassar AP, Caruso P (2018). Accuracy of SOFA, qSOFA, and SIRS scores for mortality in cancer patients admitted to an intensive care unit with suspected infection. J. Crit. Care.

[10] Moskowitz A (2017). qSOFA and SIRS as predictors of critical care intervention among patients with suspected infection. Crit. Care Med..

[11] Park HK, Kim WY, Kim MC, Jung W, Ko BS (2017). Quick sequential organ failure assessment compared to systemic inflammatory response syndrome for predicting sepsis in emergency department. J. Crit. Care.

[12] Seymour CW (2016). Assessment of clinical criteria for sepsis: For the third international consensus definitions for sepsis and septic shock (Sepsis-3). JAMA.

[13] Oken MM (1982). Toxicity and response criteria of the Eastern Cooperative Oncology Group. Am. J. Clin. Oncol..

[14] McNarry AF, Goldhill DR (2004). Simple bedside assessment of level of consciousness: Comparison of two simple assessment scales with the Glasgow Coma scale. Anaesthesia.

[15] Bedogni G, Tsybakov A, Berlin S (2009). Clinical prediction models—A practical approach to development, validation and updating. Development.

[16] Sullivan LM, Massaro JM, D'Agostino RB (2004). Presentation of multivariate data for clinical use: The Framingham Study risk score functions. Stat. Med..

[17] Khwannimit B, Bhurayanontachai R, Vattanavanit V (2018). Comparison of the performance of SOFA, qSOFA and SIRS for predicting mortality and organ failure among sepsis patients admitted to the intensive care unit in a middle-income country. J. Crit. Care.

[18] Gaini S, Relster MM, Pedersen C, Johansen IS (2019). Prediction of 28-days mortality with sequential organ failure assessment (SOFA), quick SOFA (qSOFA) and systemic inflammatory response syndrome (SIRS)—A retrospective study of medical patients with acute infectious disease. Int. J. Infect. Dis..

[19] Raith EP (2017). Prognostic accuracy of the SOFA score, SIRS criteria, and qSOFA score for in-hospital mortality among adults with suspected infection admitted to the intensive care unit. JAMA.

[20] Freund Y (2017). Prognostic accuracy of sepsis-3 criteria for in-hospital mortality among patients with suspected infection presenting to the emergency department. JAMA.

[21] Kim M (2017). Predictive performance of the quick sequential organ failure assessment score as a screening tool for sepsis, mortality, and intensive care unit admission in patients with febrile neutropenia. Support Care Cancer.

[22] Rosolem MM (2012). Critically ill patients with cancer and sepsis: Clinical course and prognostic factors. J. Crit. Care.

[23] Christodoulou C (2007). Performance status (PS): A simple predictor of short-term outcome of cancer patients with solid tumors admitted to the intensive care unit (ICU). Anticancer Res..

[24] West HJ, Jin JO (2015). JAMA oncology patient page. Performance status in patients with cancer. JAMA Oncol..

[25] Shankar-Hari M (2016). Developing a new definition and assessing new clinical criteria for septic shock: For the third international consensus definitions for sepsis and septic shock (sepsis-3). JAMA.

[26] Koozi H, Lengquist M, Frigyesi A (2020). C-reactive protein as a prognostic factor in intensive care admissions for sepsis: A Swedish multicenter study. J. Crit. Care.

[27] Vincent JL (1996). The SOFA (Sepsis-related Organ Failure Assessment) score to describe organ dysfunction/failure. On behalf of the Working Group on Sepsis-Related Problems of the European Society of Intensive Care Medicine. Intensive Care Med..

[28] Leone M (2009). Oxygen tissue saturation is lower in nonsurvivors than in survivors after early resuscitation of septic shock. Anesthesiology.

[29] Ahn S, Lee YS, Lim KS, Lee JL (2012). Emergency department cancer unit and management of oncologic emergencies: Experience in Asan Medical Center. Support Care Cancer.

[30] Camou F (2020). Long-term prognosis of septic shock in cancer patients. Support Care Cancer.

[31] Asdahl PH, Christensen S, Kjaersgaard A, Christiansen CF, Kamper P (2020). One-year mortality among non-surgical patients with hematological malignancies admitted to the intensive care unit: A Danish nationwide population-based cohort study. Intensive Care Med..

