# Supplementary material for: Development and validation of a new risk scoring system for solid tumor patients with suspected infection
Source: Sci Rep. 2022 Mar 2;12:3442. doi: 10.1038/s41598-022-07477-w (PMC8891281; doi:10.1038/s41598-022-07477-w)

Supplementary S.1. Point allocation for each factor in a new risk scoring system

|  |  | Reference value (W) | Bi | Bi (W-Wref) | B | Bi/B | Points |
| --- | --- | --- | --- | --- | --- | --- | --- |
|  |  |  |  |  | 0.612 |  |  |
| ECOG PS |  |  |  |  |  |  |  |
|  | 0-1 | 0 (ref) |  | 0 |  | 0 | 0 |
|  | 2 | 1 | 1.272 | 1.272 |  | 2.080 | 2 |
|  | **3-4** | **1** | **1.835** | **1.835** |  | **3.000** | **3** |
| SpO2  (%) |  |  | -0.106 |  |  |  |  |
|  | <94 | 89.33 |  | 0.811 |  | 1.326 | 1 |
|  | ≥94 | 96.99 (ref) |  | 0 |  | 0 | 0 |
| Creatinine  (mg/dL) |  |  | 0.452 |  |  |  |  |
|  | <1.2 | 0.75 (ref) |  | 0 |  | 0 | 0 |
|  | ≥1.2 | 2.44 |  | 0.764 |  | 1.249 | 1 |
| Total  Bilirubin  (mg/dL ) |  |  | 0.083 |  |  |  |  |
|  | <1.2 | 0.59 (ref) |  | 0 |  | 0 | 0 |
|  | ≥1.2 | 4.40 |  | 0.318 |  | 0.520 | 1 |
| CRP  (mg/dL) |  |  | 0.059 |  |  |  |  |
|  | <10.0 | 4.46 (ref) |  | 0 |  | 0 | 0 |
|  | ≥10.0 | 19.61 |  | 0.899 |  | 1.470 | 1 |
| Lactate  (mmol/L) |  |  |  |  |  |  |  |
|  | <2.0 | 0 (ref) |  | 0 |  | 0 | 0 |
|  | ≥2.0 | 1 | 0.949 | 0.949 |  | 1.552 | 2 |

Bi, raw coefficient value; B, regression coefficient of the reference predictor; W, reference value for each category, ECOG, eastern cooperative oncology group; PS, performance status; SpO_2_, peripheral oxygen saturation; CRP, c-reactive protein

Supplementary S.2. The calibration graphs between observed and predicted mortality in the development cohort (A) and the validation cohort (B).


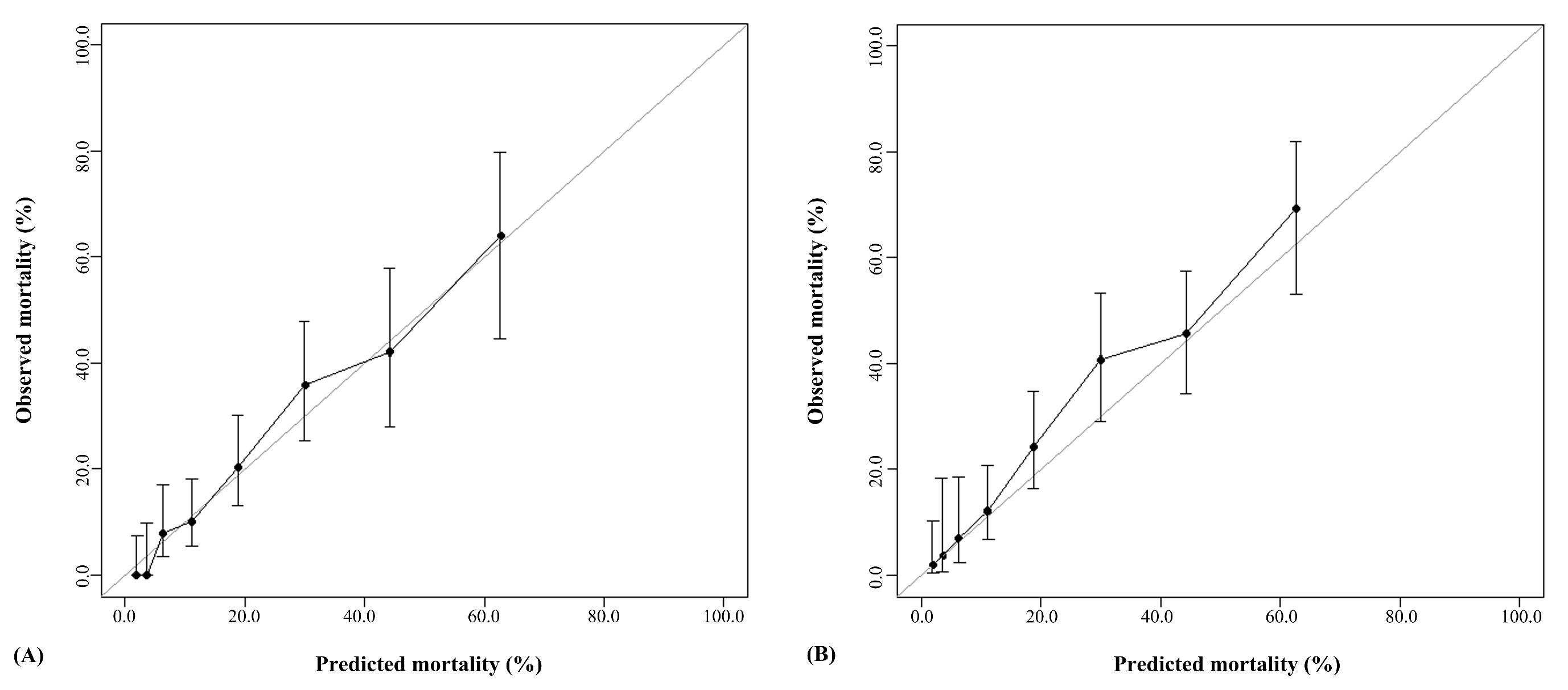


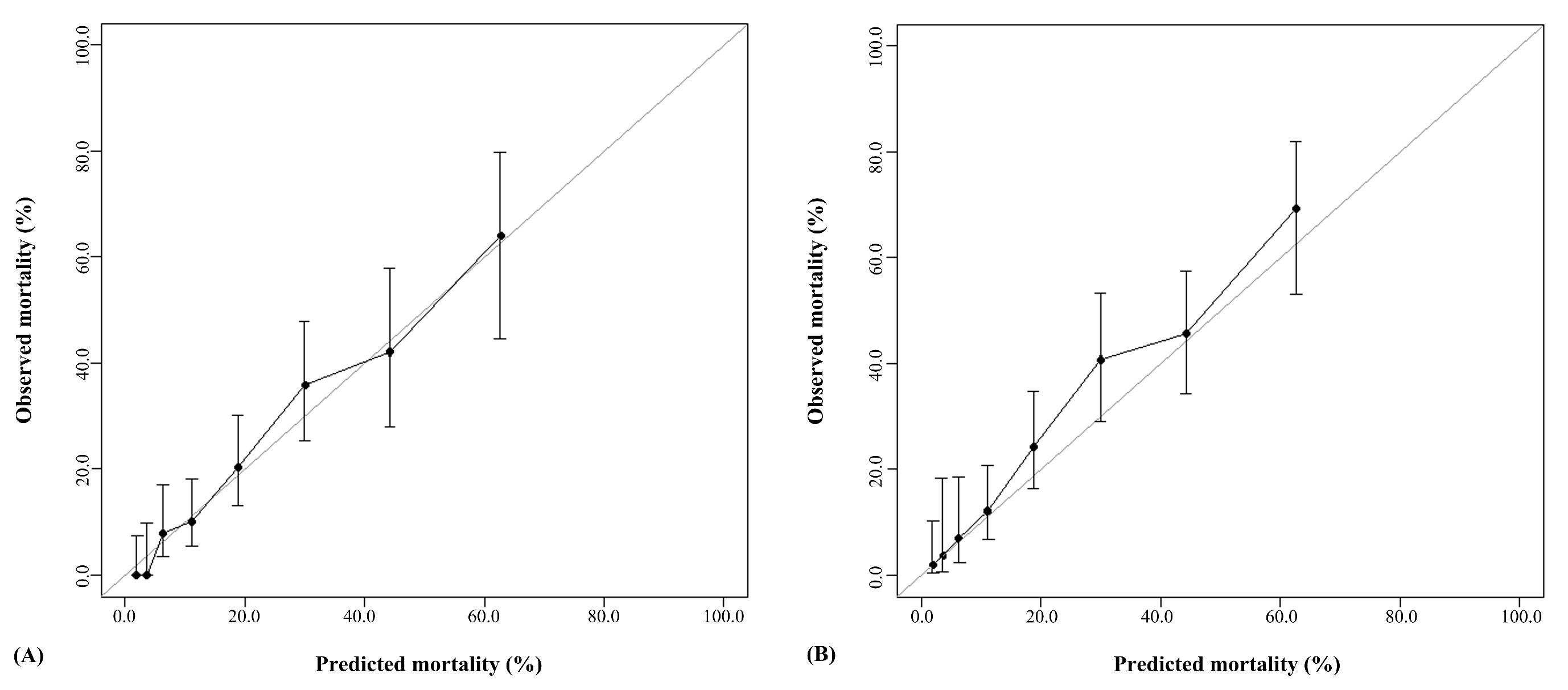

Supplement: Supplementary file 1 — Supplementary Information. [file 41598_2022_7477_MOESM1_ESM.docx]
